# Analytical and clinical validation of a novel MeltPlus TB-NTM/RIF platform for simultaneous detection of *Mycobacterium tuberculosis complex*, *Non-Tuberculous Mycobacteria* and rifampicin resistance

**DOI:** 10.3389/fcimb.2025.1534268

**Published:** 2025-02-10

**Authors:** Zhuo Wang, Yuanwu Zou, Zihan Wei, Guanghong Bai, Xiaolin Wang, Shaoyi Qu, Jie Shi, Yaping Jiang, Cuijiao Gu

**Affiliations:** ^1^ Department of Clinical Laboratory, Shaanxi Provincial Hospital of Tuberculosis Prevention and Treatment Hospital, Xian, China; ^2^ Department of Clinical Laboratory, Shaanxi Provincial People’s Hospital, Xian, China; ^3^ Department of Clinical Laboratory, Xi’an No.3 Hospital, The Affiliated Hospital of Northwest University, Xian, China

**Keywords:** tuberculosis, *Non-Tuberculous Mycobacteria*, rifampicin resistance, molecular diagnosis, MeltPlus TB-NTM/RIF

## Abstract

**Background:**

Rapid and accurate diagnosis of tuberculosis, particularly rifampin (RIF)-resistant tuberculosis (RR-TB) and *Non-Tuberculous Mycobacteria* (NTM), is essential for implementing appropriate proper therapy to benefit patients and improve TB/NTM patient management.

**Methods:**

In this study, we developed a novel MeltPlus MTB-NTM/RIF platform, designed to simultaneously detect *Mycobacterium tuberculosis complex* (MTBC), NTM and RIF resistance. The platform was evaluated for its limit of detection (LOD) and specificity before clinical validation, followed by a prospective single-center study in patients with presumptive TB cases.

**Results:**

The calculated LOD for MTBC, NTM and RIF susceptibility was found to be 10.31 CFU/mL, 57.55 CFU/mL and 48.584 CFU/mL, respectively. The assay showed a sensitivity of 98.76% (95% CI: 96.41-99.74%) and a specificity of 94.42% (95% CI: 90.82-96.92%) for MTBC detection compared to the bacteriological TB standard. For NTM detection, the assay demonstrated a sensitivity of 91.98% (95% CI: 76.32-98.14%) and a specificity of 99.59% (95% CI: 98.54-99.95%). RIF resistance detection showed a sensitivity of 90.24% (95% CI:76.87-97.28%) and specificity of 95.98% (95% CI: 91.89-98.37%), with a high level of diagnostic agreement (*Kappa*: 0.8338) compared to GeneXpert. Sanger sequencing revealed that novel assay correctly classifies 98.6% of study cases as RIF resistant or susceptible, slightly higher that of GeneXpert.

**Discussion:**

These findings indicate that the novel MeltPlus MTB-NTM/RIF platform provides a rapid and accurate method for the simultaneously detecting MTBC, NTM, and RIF resistance, making it a promising tool for clinical TB/NTM diagnosis and management, further multi-center and field studies are recommended to validate its broader applicability.

## 1 Introduction

Tuberculosis (TB) remains one of the leading infectious diseases worldwide, with an estimated 10.8 million new cases and 1.25 million deaths reported in 2023 by World Health Organization (W.H.O., 2024). Early and accurate diagnosis is crucial for effective TB control and management, particularly in regions with high disease burden. The *Mycobacterium tuberculosis complex* (MTBC) is the causative agent of TB, but the diagnosis is complicated by the presence of *Non-Tuberculous Mycobacteria* (NTM). NTMs cause similar clinical symptoms but require distinct treatment approaches, presenting a significant challenge in differentiating between the two in clinical settings. Conventional diagnostic methods, such as acid-fast bacillus smears and cultures, cannot reliably distinguish MTBC from NTM, often leading to diagnostic delays or errors. Furthermore, NTMs are increasingly recognized as clinically relevant pathogens, complicating the diagnostic landscape further. Specifically, other studies have revealed that the failure to detect NTM infections frequently results in the misdiagnosis of lung diseases with vague symptoms, leading to inappropriate and potentially harmful treatments and can potentially foster TB drug resistance (Griffith et al., 2007). Moreover, the emergence of multidrug-resistant TB (MDR-TB), with approximately 45,000 new cases reported globally in 2024, particularly resistance to rifampicin, affecting around 104,000 individuals, has further complicated TB control efforts (Lange et al., 2020). Rapid and accurate detection of both MTBC and NTM, as well as rifampicin resistance, is therefore essential for the timely initiation of appropriate treatment regimens and for controlling TB spread.

Traditional diagnostic methods, such as acid-fast bacillus smears (AFB) and cultures, remain widely used but are limited in their effectiveness. Specifically, AFB smears suffer from low sensitivity and cultures are constrained by prolonged turnaround times (Parsons et al., 2011). To overcome these challenges, fluorescence microscopy has emerged as a promising alternative. By enhancing the visibility of bacilli, it offers approximately 10% greater sensitivity compared to traditional AFB smears (Albert et al., 2010; Mugenyi et al., 2024). Moreover, advancements in culture techniques have introduced liquid culture methods, which significantly shorten detection times to 10~14 days, compared to the 2~4 weeks typically required for traditional solid media. These improvements represent significant steps forward, but limitations such as reliance on specialized equipment and longer processing times compared to newer technologies remain. In contrast, molecular diagnostic tools have revolutionized TB diagnostics by not only rapid and accurate detection but also the ability to identify drug resistance patterns within several hours (Sekyere et al., 2019). GeneXpert MTB/RIF assay, endorsed by the WHO, is widely used to detect MTBC and rifampicin resistance directly from clinical specimens within two hours. However, despite its high sensitivity and specificity, GeneXpert MTB/RIF has some limitations, including potential false-positive results for rifampicin resistance and the inability to distinguish between MTBC and NTM (McNerney and Zumla, 2015). False positive results might derive from technical issues such as probe binding delays or the use of specific probes (e.g., probe B) in the GeneXpert assay (Berhanu et al., 2019). Probe binding delays can cause the assay to misinterpret the presence of resistance, particularly when the cycle threshold values are low. On the other hand, the presence of heterogeneous mutations can lead to challenges in accurately identifying the target sequences, thereby reducing the specificity of the assay (Van Rie et al., 2020). In response to these limitations, various other commercial PCR kits have been developed to enhance the detection capabilities for TB diagnostics. These kits aim to offer comprehensive diagnostic information, including the differentiation between MTBC and NTM and detection of rifampicin resistance (Lee et al., 2018; Shin et al., 2020; Fan et al., 2023). Unfortunately, these commercial kits need to be used in combination for these purposes, in other words, there are fewer reports of achieving detection of these targets in the same tube. Given these limitations, there is a clear need for a more efficient, centralized assay that can detect MTBC, NTM and rifampicin resistance in a single tube to streamline the diagnostic process, reduce turnaround time, and lower the costs associated with TB diagnostics.

In the current, we aimed to develop and validate a novel centralized assay, based on asymmetric PCR combined with melting curve analysis, for simultaneous diagnosis of MTBC, NTM and rifampicin resistance in presumptive TB patients at a single center. Specifically, we compare the diagnostic accuracy, sensitivity, and specificity of MeltPlus MTB-NTM/RIF against the GeneXpert MTB/RIF and a commercial PCR kit. By providing a detailed comparative analysis, this study seeks to contribute to the optimization of TB diagnostic strategies, ultimately enhancing patient outcomes and supporting global TB control efforts, particularly in high-burden settings where rapid and accurate diagnostics are critical for effective disease control.

## 2 Methods

### 2.1 Asymmetric PCR-combined MCA assay development

#### 2.1.1 Primer and probe design

Two highly conserved regions, including Insertion Sequence (*IS*) 6110 and gyrB, were selected to design primers and probes for detection of MTBC (Chaudhari et al., 2016; Chen et al., 2021). Detection of NTM was achieved by targeting the *16S* rRNA gene that present in all mycobacterial species, while the target region of *IS* 6110 and gyrB are not present in the NTM species (Uwamino et al., 2023). For rifampicin resistance, 81bp region of the *rpoB* gene, which is crucial for determining rifampicin resistance, is utilized for designing specific primers and probes. Additionally, primers targeting the human tRNA-processing ribonuclease P (RNase P) gene were also included in this study, added as the extraction and amplification control.

#### 2.1.2 PCR amplification and muti-color melting curve analysis

The PCR amplification was performed on the SLAN-96S real-time PCR system (Hongshi Tech Co., ltd, China). Amplification was carried out in 25 μL reaction volumes, including 1×Taq HS Buffer (Mg ^2+^ plus) (Nanjing Vazyme, China), 0.1 U/μL Taq HS DNA polymerase (Nanjing Vazyme, China), limiting and excess primers, Taqman probes and 5 μL template. The detailed concentration of primers and probes are presented in **Supplementary Table S1**. PCR amplification was performed under the following conditions: 95°C for 10 min for initial denaturation, followed by 13 touch down cycles of denaturation at 95°C for 25 s, annealing and extension at 72°C for 30 s (-1°C/cycle). And then 38 cycles of 95°C for 25 s, annealing at 58°C for 30 s, followed by extension at 72°C for 30 s. Melting curve analysis was initiated with a denaturation step of 1 min at 95°C, followed by hybridization for 1 min at 45°C. The temperature was gradually increased from 45°C to 90°C at a rate of 0.04°C/s, with fluorescence signals acquired in the FAM, VIC, ROX and Cy5 channel, allowing for the identification of specific melting peaks corresponding to each target. Double distilled water was added to the tube to serve as the negative control.

#### 2.1.3 Sample processing and DNA extraction

DNA was extracted from the sputum or bronchoalveolar lavage fluid (BALF) sediments using EX-TB DNA extraction kit and GeneFlex 16 Fully automated nucleic acid extraction instrument (Tianlong Tech Co., Ltd.) according to manufacturer’s instructions. Briefly, the 1.0 mL of raw sputum or BALF was pipetted to 2mL N-acetylL-cysteine-2% NaOH, vortexed thoroughly and then incubated for 15 min. Subsequently, 1 mL of the liquefied sample was added the sample loading well. DNA extraction carried out using a magnetic bead-based automatic extraction protocol, and the extracted DNA was used as a template for PCR amplification. For the GeneXpert MTB/RIF assay, 1.0 mL of raw sediments was added to 2.0 mL of the liquefying agent according to the manufacturer’s instructions. After incubation for 15 min, 2.0 mL of this mixture was pipetted to cartridge and it was loaded subsequently in GeneXpert instrument.

### 2.2 Analytic evaluation of the assay

The clinical non-infected sputum samples, confirmed to be negative for MTBC and NTM by Mycobacteria Growth Indicator Tube (MGIT) liquid medium inoculation, were selected as model to further evaluation the performance of the established platform. The samples artificially spiked with a known concentration of the reference strain MTB H37Rv at series concentrations, 1 CFU/mL to 500 CFU/mL for MTBC, 10 CFU/mL to 2000 CFU/mL for NTM and rifampicin susceptibility. Each dilution was prepared and tested in twenty replicates to ensure statistic reliability. Negative controls (non-infected sputum samples without bacterial spiking) and positive controls (samples spiked with concentrations well above the detection threshold) were included in each assay run to validate performance. The lower limit of detection (LOD) was determined using probit analysis, defined as the concentration of CFU/mL at the lowest dilution which yield the detection of the targets ≥95% probability. Additionally, the analytical specificity of the novel platform was tested using other respiratory bacterial cultures, of which concentration ≥10^6^ CFU/mL, and some commonly NTM isolates are also used to verify the inclusiveness of developed assay (**Supplementary T** **able S2**).

### 2.3 Ethical approval statement

Informed consent was obtained from all subjects, and the study was approved by the Institutional Review Board of Shaanxi Provincial Hospital of Tuberculosis Prevention and Treatment Hospital (Ethics approval number: 2024No.26). This approval was in line with the Helsinki declaration as revised in 2013 and its later amendments.

### 2.4 Study participants and procedure

In this prospective single-center study, sputum and BALF specimens were collected from 534 presumptive TB cases (between March 2024 and July 2024) following testing with smear microscopy, mycobacterial culture and GeneXpert MTB/RIF (Cepheid Inc., USA) at the clinical laboratory of Shaanxi Provincial Hospital of Tuberculosis Prevention and Treatment Hospital, Xian, China. Sputum and BALF sediments, whether TB positive or negative, regardless of culture status and GeneXpert RIF resistance results, were included in the study. Each specimen was assigned a unique study number, and patient personal information were removed.

### 2.5 Acid-fast bacillus smears and mycobacterial culture

Samples were smeared onto glass slides, air-dried, and heat-fixed. The smears were then stained using the modified Ziehl-Neelsen staining method. Slides were immersed in carbol fuchsin, decolorized with acid alcohol, and counterstained with methylene blue. After thorough washing, the slides were examined under a light microscope using oil immersion (1000× magnification).

Sputum and BALF were decontaminated using the N-acetyl-L-cysteine (NALC)-NaOH method. The treated samples were then concentrated by centrifugation and inoculated onto both solid (Löwenstein-Jensen medium) or liquid media (MGIT liquid culture system). Solid media were incubated at 37°C and inspected weekly for colony formation for up to 6~8 weeks. Liquid cultures were monitored using an automated detection system, with positive results typically observed within 2~6 weeks. Colony morphology and growth characteristics on the media provided initial identification of the mycobacterial species (Den Hertog et al., 2010; Watanabe Pinhata et al., 2018), which was further confirmed by molecular or biochemical tests (Jung et al., 2016; Kuentzel et al., 2018; Lyamin et al., 2023).

### 2.6 Statistical analysis

Data were analyzed statistically using IVD Statistics and GraphPad Prism 8.0.2. Sensitivity, specificity, accuracy and their confidence intervals of the assay were calculated by comparing the MeltPlus TB-NTM/RIF with those obtained with the reference methods, including culture, GeneXpert MTB/RIF and a commercial PCR kit (*Mycobacterium* Real Time PCR Detection Test, CapitalBio Tech Co., Ltd. China).

## 3 Results

### 3.1 Workflow of the established assay

**Figure 1:**
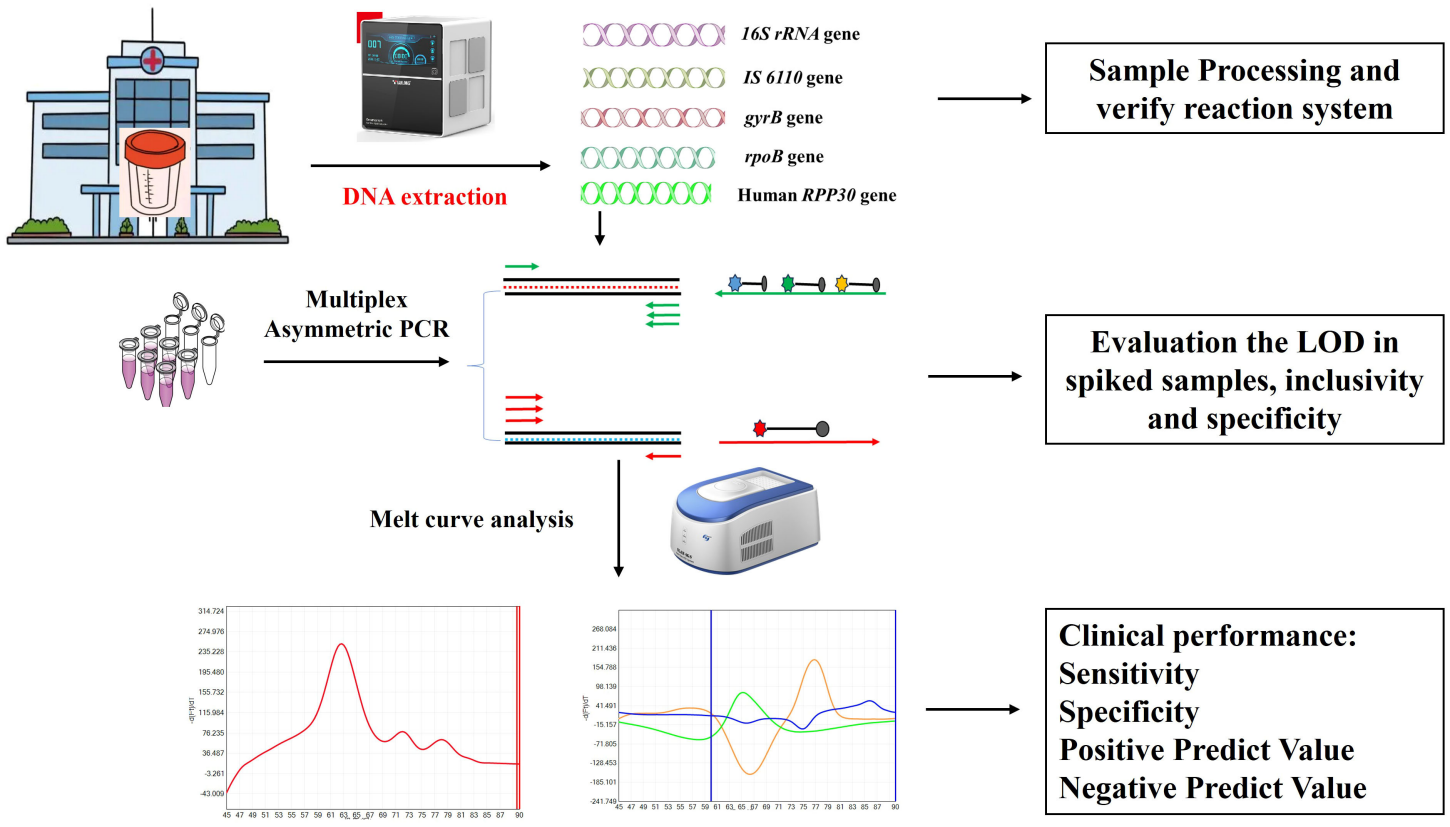
Schematic illustration of MeltPlus TB-NTM/RIF platform.

The present study has developed a novel platform that enables the simultaneous diagnosis of MTBC/NTM and rifampicin resistance in a single tube, offering both rapidity and cost-efficiency. As shown in **Figure 1**, the platform is comprised of two distinct components: sample processing and PCR amplification with subsequent melting curve analysis, in which the former includes sputum/BALF liquefaction and fully automated nucleic acid extraction. After melting curve analysis, the actual Tm values of the probes ranged from 61°C to 84°C, and each target can be differentiated based on their distinct Tm value and fluorophore (**Figure 1**). A test was considered positive for MTBC if it was positive for *IS 6110*/*gyrB* and *RNase P* genes, which Tm values were found to be 63 ± 1°C and 72 ± 1°C in Cy5 channel, respectively. A test was considered positive for NTM if it was positive for *16s RNA* and *RNase P* genes, while negative for *IS 6110/gyrB*, which Tm values were found to be 77 ± 1°C and 72 ± 1°C in Cy5 channel, respectively. Three different Tm peaks, in respect to 83 ± 1°C in FAM channel, 63 ± 1°C in HEX channel and 75 ± 1°C in Texas red channel, would be obtained and it presented that there was no mutation in the 81bp-core region of *rpoB* gene. If ΔTm of any of the three fluorescence channels were greater than 1.5°C, it is considered that there is a mutation in the drug resistance determination area of *rpoB* and the bacteria is resistant to rifampicin.

### 3.2 LOD and specific evaluation

**Figure 2:**
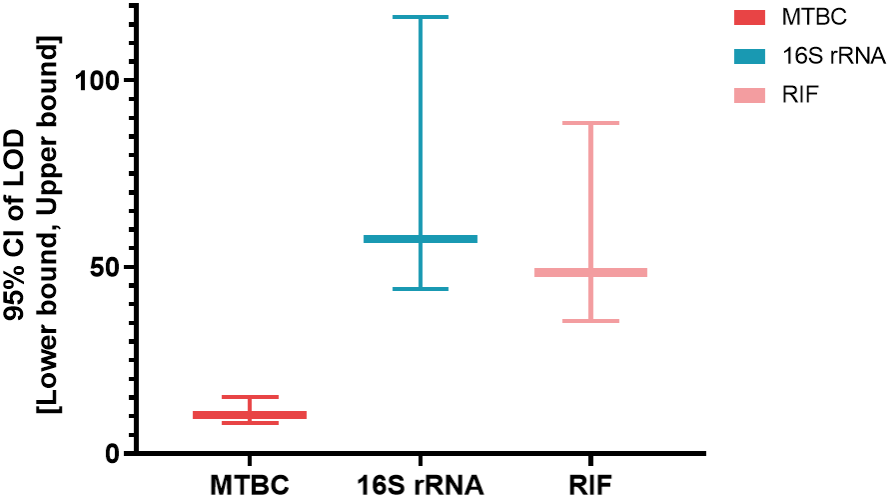
The LOD [95% CI: (Lower bound, upper bound)] of the assay for the targeted genes.

Artificially contaminated sputum samples were prepared by adding varying concentrations of MTB H37Rv to the matrix (from 1 CFU/mL to 2000 CFU/mL), and the assay was then tested on 20 replicates of each concentration to determine the LOD. The results showed that 95% (19/20) of tested 20 samples were successfully detected by MeltPlus TB-NTM/RIF down to dilutions to 10 CFU/mL for MTBC spiked samples. The mycobacterial strains were correctly 100% detected by the specific melting peak of 16S rRNA at 100 CFU/mL (20/20), while only 80% (16/20) at 50 CFU/mL and 35% (7/20) at 10 CFU/mL, respectively. All tested 20 samples successfully detected (100%) of RIF susceptibility up to 100 CFU/mL, while 75% (15/20) for 50 CFU/mL and 48.5% (9/20) for 10 CFU/mL, respectively (**Table 1**). Therefore, the calculated LOD of MeltPlus TB-NTM/RIF by probit analysis for detection of MTBC, NTM and RIF susceptibility in spiked samples was found to be 10.31 CFU/mL (CI: 8.18-15.23), 57.55 CFU/mL (CI: 44.18-117.03) and 48.584 CFU/mL (CI: 35.48-88.61), respectively (**Figure 2**).

**Table 1:** Limit of detection of the novel platform based on asymmetric PCR-combined MCA assay.

CFU/mL	IS 6110+gyrb for MTBC	16S rRNA for all Mycobacteria	rpob for rifampicin
2000	ND	20/20(100%)	20/20(100%)
1000	ND	20/20(100%)	20/20(100%)
500	20/20(100%)	20/20(100%)	20/20(100%)
200	20/20(100%)	20/20(100%)	20/20(100%)
100	20/20(100%)	20/20(100%)	20/20(100%)
50	20/20(100%)	16/20(80%)	18/20(90%)
25	20/20(100%)	13/20(65%)	14/20(70%)
10	19/20(95%)	7/20(35%)	9/20(48.5%)
5	11/20(55%)	0/20(0)	2/20(10%)
1	4/20(20%)	0/20(0)	0/20(0)

ND, Not detected

Five clinical MTBC strains, 10 NTM species and 6 non-TB bacteria were used to verify the inclusivity and specificity of the novel developed assay. The new assay correctly detected all MTBC and 10 NTM species, whereas no cross-reactivity against 6 other respiratory pathogens and distilled water, indicating that the established assay presented high specificity and could be used for clinical evaluation (**Supplementary T** **able S2**).

### 3.3 Assay performance with clinical samples

#### 3.3.1 Patient characteristics

**Figure 3:**
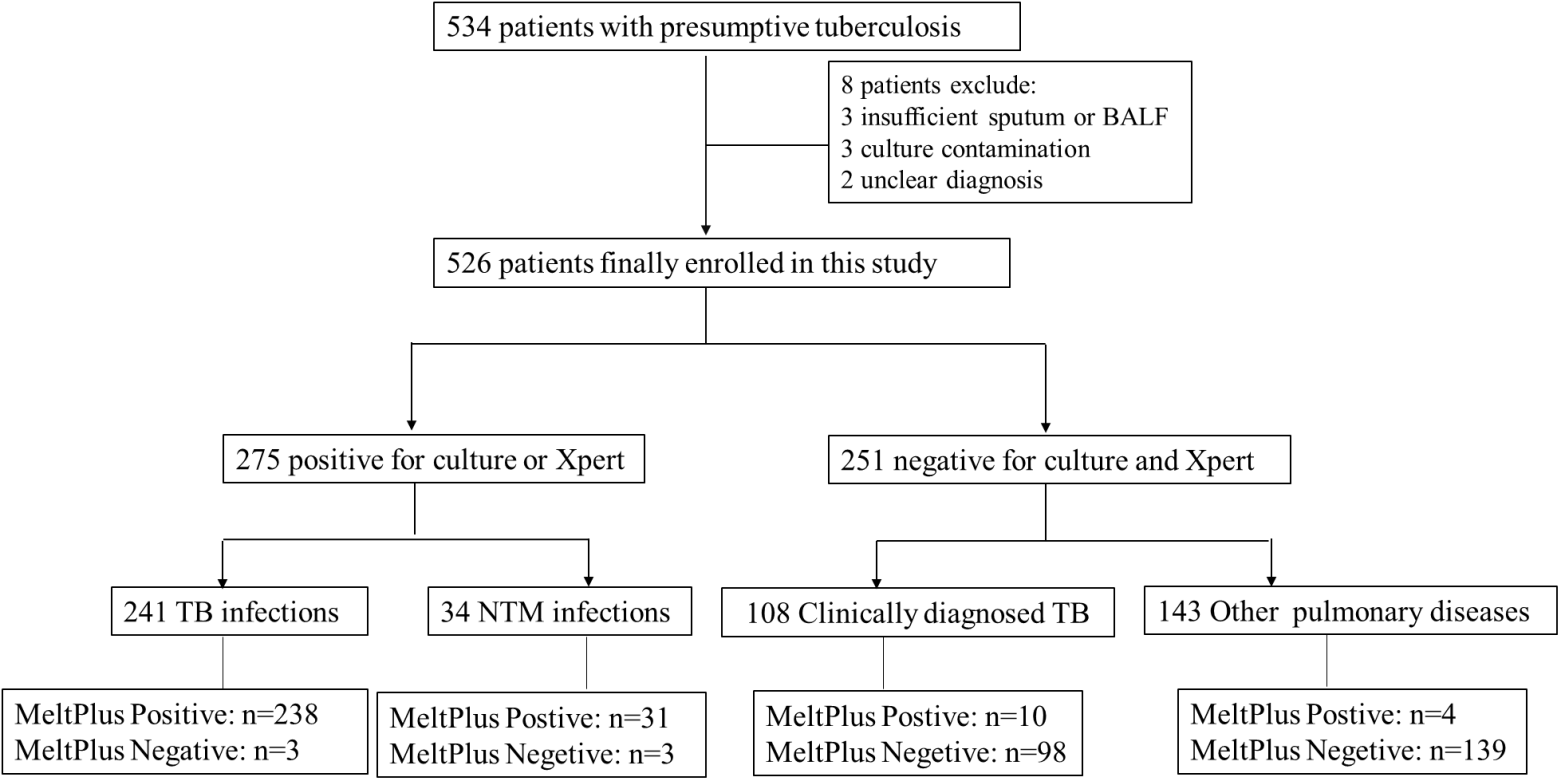
Study participants’ and exclusions from analysis eligibility.

To conduct a clinical feasibility evaluation of MeltPlus TB-NTM/RIF, a total of 534 individuals were initially included in this study. Eight individuals were excluded due to following reasons: 3 cases of insufficient sputum or BALF, 3 cases of culture contamination, and 2 cases of unclear diagnosis. Consequently, a total of 526 patients were finally included in the study (as shown in **Figure 3**). Among them, 383 (72.8%) were diagnosed with PTB or NTM pulmonary disease, including 275 confirmed as *Mycobacteria* infection through culture or GeneXpert (241 TB infections and 34 NTM infections), and 108 clinically diagnosed TB. The remaining 251 (47.7%) were negative for both culture and GeneXpert tests. The other characteristics of these patients were summarized in **Table 2**.

**Table 2:** Demographic and clinical characteristics of the 526 enrolled suspected TB patients.

Characteristics N=526	No. positive for TB N=241(%)	No. (%) positive for NTM N=34(%)	Others N=251(%)
Gender			
Male	170 (70.54%)	26 (76.47%)	136 (53.78%)
Female	71 (29.47%)	8 (23.53%)	115 (45.82%)
Age group			
≤35	49 (20.33%)	6 (17.65%)	61 (24.30%)
35-70	145 (60.17%)	23 (67.65%)	159 (63.35%)
≥70	47 (19.50%)	5 (14.71%)	31 (12.35%)
Sample types			
Sputum	198 (82.16%)	29 (85.29%)	143 (56.98%)
BALF	43 (17.84%)	5 (14.71%)	108 (3.02%)
AFB			
Positive	135 (56.02%)	19 (55.88%)	0
Negative	106 (43.98%)	15 (44.12%)	251 (100%)
Mycobacterial culture			
Positive	212 (87.97%)	34 (100%)	0
Negative	29 (12.03%)	0	251 (100%)

#### 3.3.2 Accuracy of MTBC diagnostic

Results of the comparison between MeltPlus TB-NTM/RIF and bacteriologically TB standard are shown in **Table 3**, sensitivity and specificity of the platform was found to be 98.76% (238/241; 95% CI: 96.41%-99.74%) and 94.42% (237/251; 95% CI: 90.82%-96.92%), respectively. The specificity calculation did not include the NTM cases detected. Of the participants, a total of 17 specimen showed discordant results between bacteriologically TB standard and MeltPlus TB-NTM/RIF. Among the 241 TB confirmed patients, 215 were found to be positive for GeneXpert sore use, yielding a sensitivity of 89.2% (95% CI: 85.28%-93.11%). Compared with bacteriologically TB standard, the sensitivity of GeneXpert is significantly lower than that of MeltPlus TB-NTM/RIF (P<0.001), and this might result from LOD difference and the nonhomogeneous nature of sputum or BALF (Kennedy et al., 1994). On the other hand, 14 cases without laboratory evidence presented MTBC-positive results, of which 10 cases fulfilled the criteria for clinically diagnosed TB. Therefore, the sensitivity and specificity of novel assay was 71.06% (248/349; 95% CI: 65.99%-75.76%) and 97.18% (138/142; 95% CI: 92.94%-97.18%) when compared with clinically diagnosed results.

**Table 3:** Diagnostic performance of MeltPlus TB-NTM/RIF for the detection of MTBC and NTM from 526 sputum and BALF specimens.

Analyte	The novel assay	Reference		Sensitivity % (95% CI)	Specificity % (95% CI)	OPA^e^ % (95% CI)	Cohen’s κ
		P^c^	N^d^				
MTBC^a^	P^c^	238	14	98.76 (96.41-99.74)	94.42 (90.82-96.92)	96.54 (94.53-97.97)	0.9309
	N^d^	3	237				
NTM^b^	P^c^	31	2	91.98 (76.32-98.14)	99.59 (98.54-99.95)	99.05 (97.80-99.69)	0.9203
	N^d^	3	490				

a For MTBC detection, the Reference method was microbiological reference standard, including GeneXpert and culture.

b For NTM detection, the results was compared with a commercial PCR kit.

c P, positive.

d N, negative.

e OPA, overall percent agreement.

#### 3.3.3 Diagnostic performance for NTM

Out of 526 clinical respiratory samples, 34 samples were positive for NTM using the *Mycobacterium* Real Time PCR Detection Test Kit (CapitalBio Tech Co., Ltd. China). After retrospectively reviewed the patients’ case information, we confirmed that all 34 cases fulfilled the definition of pulmonary NTM disease, with NTM detected in at least two respiratory samples collected at different times, along with the presence of respiratory symptoms similar with TB/NTM infection. Of these 34 cases, 31 were accurately identified by MeltPlus TB-NTM/RIF, while the remaining 3 samples were identified as MTBC (**Table 3**). This specimen exhibited a weakly positive result for NTM in the commercial PCR kit (with Ct value of *Mycobacterium* gene: 38.11, 36.88, 37.42 and Ct value less than 40 regarded as positive), whereas our assay result was positive for MTBC (**Supplementary T** **able S3**). This discrepancy may be attributed to the inconsistency of the LOD values, as LOD of the commercial PCR kit reported to be 5×10^3^ CFU/mL. Among the samples that were negative by both culture and GeneXpert, 2 samples were identified as NTM by MeltPlus TB-NTM/RIF, whereas the commercial PCR kit returned negative results. Therefore, MeltPlus TB-NTM/RIF showed sensitivity and specificity of 91.98% (95% CI: 76.32%-98.14%) and 99.59% (95% CI: 98.54%-99.95%) for directly detection of NTM in clinical sputum and BALF specimens.

#### 3.3.4 Performance for RIF resistance detection

Among 41 patients diagnosed with RIF-resistant by GeneXpert, 37 patients were correctly diagnosed by MeltPlus TB-NTM/RIF with a sensitivity of 90.24% (95% CI: 76.87%-97.28%). In addition, 167 of 174 patients diagnosed as RIF sensitive by GeneXpert were confirmed by MeltPlus TB-NTM/RIF, demonstrating a specificity of 95.98% (95% CI: 91.89%-98.37%). Kappa analysis was conducted to evaluate the consistency between MeltPlus TB-NTM/RIF and GeneXpert in detecting rifampicin susceptibility, yielding a *Kappa* value of 0.8338, which suggests a high level of diagnostic agreement (**Table 4**). Out of the 215 samples subjected to resistance analysis, 11 samples presented inconsistent results between the novel platform and GeneXpert, of which 4 cases of GeneXpert were diagnosed as RIF resistance and 7 cases were diagnosed as RIF sensitive. We further sequenced the PCR amplified products from the 11 clinical samples to identify presence or absence of the mutations. Results of sanger sequencing revealed that 3 samples of the former cases were diagnosed as RIF sensitive, consistent with MeltPlus TB-NTM/RIF, and 1 sample was diagnosed as RIF resistance. Two patients’ samples of the 7 cases were found to be RIF sensitive while 5 cases were found to be RIF resistance by sanger sequencing, respectively. Therefore, MeltPlus TB-NTM/RIF and GeneXpert correctly classifies 98.6% and 96.3% of study cases as RIF resistant or susceptible, respectively. And we speculate that sensitivity and specificity of the novel assay for detection of RIF susceptibility are slightly higher than those of GeneXpert.

**Table 4:** Diagnostic accuracy of MeltPlus TB-NTM/RIF for RIF susceptibility compared with GeneXpert.

The novel assay	GeneXpert		Sensitivity % (95% CI)	Specificity % (95% CI)	OPA^c^ % (95% CI)	Cohen’s κ
	R^a^	S^b^				
R^a^	37	7	90.24 (76.87-97.28)	95.98 (91.89-98.37)	94.88 (91.03-97.42)	0.8338
S^b^	4	167				

a R, rifampin resistant.

b S, rifampin susceptible.

c OPA, overall percent agreement.

## 4 Discussion

MeltPlus TB-NTM/RIF, integrating the detection of MTBC, NTM and rifampicin resistance into one test, can significantly improve the management of TB or NTM infections, particularly in high-burden settings where rapid and accurate diagnostics are critical for effective disease control. The platform delivers results within 3 hours (from sample to answer) and costs approximately $8 per sample (including nucleic acid extraction), making it a highly cost-effective alternative. In comparison, the commercial Xpert MTB/RIF test costs approximately $65 per sample, as the negotiated lower price is not applicable in China. By integrating the detection of multiple targets into a single assay, the MeltPlus platform also reduces the need for separate tests, further minimizing costs and improving operational efficiency in laboratories with limited resources. These features collectively position the platform as a scalable and practical solution for TB and NTM management in regions with high disease burden and constrained healthcare infrastructure.

The novel centralized platform was further validated and demonstrated to be highly accurate, sensitive and reliable. LODs for MTBC was found to be 10.31CFU/mL, with the sensitivity surpasses the GeneXpert’s assay (131CFU/mL) and are comparable to the results for Xpert Ultra (15.6 CFU/mL), iFIND TBR (13.34 CFU/mL) and InnowaveDX MTB/RIF(9.6 CFU/mL) (Helb et al., 2010; Chakravorty et al., 2017; Deng et al., 2023; Ou et al., 2024). The assay also detected MTBC at 100 CFU/mL with 100% accuracy, outperforming some traditional culture methods that typically require higher bacterial loads for reliable detection. The analytical performance for NTM and RIF susceptibility is lower at a detection limit of 50 CFU/mL, but it remains comparable to the performance reported for the commercial PCR kit and GeneXpert assay. The increased sensitivity of MeltPlus TB-NTM/RIF undoubtedly assisted diagnosis and guide treatment decision for pulmonary TB.

Clinical validation of MeltPlus TB-NTM/RIF platform involved a comprehensive study with 526 patients, where the assay exhibited remarkable sensitivity (98.76%) and specificity (94.42%) for detecting MTBC. This high performance is comparable to, and in some cases exceeds, that of traditional diagnostic methods. For example, the sensitivity of platform exceeds that of the GeneXpert assay, which demonstrated a sensitivity of 89.2% in our study. This finding is consistent with other reports, wherein the sensitivity of the GeneXpert for tuberculosis diagnosis has been reported to 83% to 90% (Boehme et al., 2010). Increased sensitivity to benefit those at risk for false negatives may reduce the specificity, leading to a higher chance of false positives (Deng et al., 2023). As we evaluated the clinical performance of the new method for the detection of MTBC and NTM, 10 of the 16 samples without TB and NTM etiology met the criteria for clinical diagnosis of TB, but the remaining 6 would be considered as false positives, and these false positives may occur attributable to the sample cross contamination. Pre-PCR sample processing can produce many aerosols, particularly in labs with numerous *Mycobacterial* samples, potentially leading to false positives of MeltPlus MTBC-NTM/RIF in the present study (Mifflin, 2007). On the other hand, increased false-positive results from ultrasensitive molecular assays have also been reported in other studies, highlighting a common challenge with such highly sensitive diagnostic tools. For example, as demonstrated by a study conducted by Zhang et al., the use of Xpert Ultra for tuberculosis diagnosis led to a higher rate of false positives, which the authors attributed to the assay’s ability to detect minute amounts of DNA that may not necessarily indicate active infection (Zhang et al., 2020). Similarly, Johnson et al. found that, in patients who had previously undergone treatment for TB, an ultrasensitive assay for detecting MTBC produced false-positive results, likely due to residual DNA from dead bacteria (Boyles et al., 2014). Therefore, clinicians should interpret the results from the MeltPlus TB-NTM platform within the broader clinical context, especially for the patients with a history of TB or NTM infection as the presence of residual DNA from non-viable bacteria might lead to false-positive results.

NTM infections contribute to substantial morbidity and mortality globally, it is not routinely diagnosed despite the availability of treatment in many developing countries (Lange et al., 2020). However, physicians, in these resource-limited regions, often initiate presumptive treatment, which can be toxic and time-consuming (Sarro et al., 2021). Identifying these patients and ensuring appropriate treatment is critical to combat TB drug resistance and effectively treat those with NTM infection or TB. The novel platform presented good sensitivity (91.98%) and specificity (99.59%) for NTM detection in sputum and BALF specimens. We found that the sensitivity of our platform was slightly lower than that of commercial PCR kit in this study, however, the commercial kit’s sensitivity for detecting MTBC is relatively low, making it difficult to accurately confirm the validity of our detection results. Unfortunately, we were unable to conduct sanger sequencing to verify the results due to the low bacterial loads. Despite the limitations, we successfully achieved simultaneous detection of MTBC, NTM and RIF resistance in a single tube.

The centralized platform also demonstrated superior sensitivity and specificity compared to the GeneXpert MTB/RIF assay for detection of rifampicin resistance. In our study, discrepancies between two methods were observed in 11 cases. Subsequent analysis using Sanger sequencing revealed that our platform exhibited a higher concordance with sequencing results (72.7% *vs* 27.3%), with 2 cases were defined as false positive and 1 case was defined as false negative. This suggests that our assay may offer improved accuracy in detecting RIF resistance, particularly in cases where the GeneXpert assay may produce false-positive or false-negative results. These findings are especially significant in clinical settings, where precise detection of rifampicin resistance is crucial for the appropriate management of TB. Accurate identification of drug-resistant strains directly influences treatment decisions and patient outcomes. Misidentification of rifampicin resistance could lead to the use of ineffective treatment regimens, potentially contributing to the development of multidrug-resistant TB (MDR-TB) (Makhado et al., 2018).

The evaluation of diagnostic kits for detecting MTBC, NTM, and rifampicin resistance is crucial for improving the management and control of TB and NTM infections. Various nucleic acid amplification tests (NAATs) have been developed to enable rapid and accurate detection of these targets. Among these, the COBAS Amplicor MTB, COBAS TaqMan MTB, and AdvanSure TB/NTM real-time PCR kits are widely used in clinical settings. The COBAS Amplicor MTB assay, while effective, has limitations in specificity, particularly in samples with low optical density, leading to false-positive outcomes (Kim et al., 2011). To address these issues, the COBAS TaqMan MTB assay, which replaced the Amplicor version, demonstrated improved performance with a sensitivity of 88.4% and a specificity of 98.8% for respiratory specimens (Bloemberg et al., 2013). Similarly, the AdvanSure TB/NTM real-time PCR kit was evaluated for its ability to differentiate between MTBC and NTM, showing a sensitivity of 76.7% and a specificity of 99.7% for MTBC detection. In comparison, the MeltPlus MTB-NTM/RIF platform exhibited a significantly higher sensitivity (98.76%) for detecting MTBC. While its specificity was slightly lower than the COBAS TaqMan MTB and AdvanSure TB/NTM assays, it remained within a comparable range, highlighting its reliability in MTBC diagnosis. For NTM detection, the AdvanSure TB/NTM kit achieved a sensitivity of 73.9% and a specificity of 100%, reflecting its high accuracy (Kim et al., 2020). The MeltPlus platform demonstrated slightly lower but comparable sensitivity and specificity, making it a competitive option for NTM diagnosis.

Beyond MTBC and NTM detection, the MeltPlus platform also shows promise in rifampicin resistance diagnosis. It achieved a combined sensitivity of 97.62% (in conjunction with Sanger sequencing: 41/42, 95% CI: 87.43–99.94%), which is slightly lower than BD’s Max MDR-TB (99.1%) and iFIND TBR (98.15%), comparable to Bruker/Hain’s FluoroType MTBDR (97%), and superior to Roche’s cobas MTB-RIF/INH (91%), Abbott’s RealTime MTB RIF/INH (94%), and InnowaveDX MTB/RIF (86.4%) (Deng et al., 2023; Xie et al., 2024). Although the clinical performance of the MeltPlus platform is influenced by factors such as operator variability, population differences, and reagent performance, the current findings suggest that it offers a competitive edge over its counterparts. With its high sensitivity for MTBC detection, reliable performance in NTM detection, and competitive rifampicin resistance diagnosis capabilities, MeltPlus MTB-NTM/RIF platform demonstrates significant potential for enhancing TB and NTM management in clinical practice.

Despite the promising results, some limitations should be noted. The assay’s performance in detecting low bacterial loads, particularly for NTM, warrants further investigation. Furthermore, the assay’s validation has so far been limited to a relatively narrow range of clinical samples and settings. To confirm its generalizability and robustness, it is essential to expand validation efforts to include a broader array of clinical specimens from diverse patient populations and various geographical regions. This comprehensive validation would help ensure that the assay performs consistently and reliably across different clinical contexts, thus supporting its potential application in routine practice. Additionally, the novel platform cannot accurately detect samples co-infected with MTBC and NTM, the platform tends to misdiagnose the samples as solely MTBC infections. A previous multicenter clinical study in China revealed that the co-infection rate of MTBC and NTM to be approximately 1.2% (Wang et al., 2023). Although this prevalence is relatively low, these co-infection cases pose specific challenges for clinicians in developing effective treatment plans.

## 5 Conclusion

The diagnostic accuracy of MeltPlus TB-NTM/RIF platform for the detection of MTBC, NTM and rifampicin resistance was highly concordant with that of reference method (Overall percent agreement >95%, *Kappa* value >0.75). It’s enhanced sensitivity, specificity and diagnostic accuracy, coupled with the convenience of simultaneous testing, make it a valuable addition to the current diagnostic toolkit for TB/NTM infections. Future studies should focus on validating these findings in larger and more diverse patient populations to further establish the platform’s clinical utility. Furthermore, with the development of the isoniazid (INH) detection system, the novel platform is also expected to effectively detection INH resistance, further expanding its clinical applications and enhancing its utility in guiding MDR-TB treatment strategies.

## Data Availability

The original contributions presented in the study are included in the article/**Supplementary Material**. Further inquiries can be directed to the corresponding author.
