# Supplementary material for: Analytical and clinical validation of a novel MeltPlus TB-NTM/RIF platform for simultaneous detection of Mycobacterium tuberculosis complex, Non-Tuberculous Mycobacteria and rifampicin resistance
Source: Front Cell Infect Microbiol. 2025 Feb 10;15:1534268. doi: 10.3389/fcimb.2025.1534268 (PMC11847904; doi:10.3389/fcimb.2025.1534268)
Supplement: Supplementary file 1 [file Table1.docx]

**Table S1 Reaction mix composition for asymmetric PCR-combined MCA assay**

| **Component** | **Final Concentration** |
| --- | --- |
| 10 × Taq HS Buffer (Mg ^2+^ plus) | 1X |
| dNTP Mix (10 mM each) | 0.2 mM each |
| Taq HS DNA polymerase (5 U/μl) | 0.1 U/μL |
| MTBC *IS6110* gene forward primer | 0.2 μM |
| MTBC *IS6110* gene reverse primer | 1.6 μM |
| MTBC *IS6110* -Cy5 probe | 0.2 μM |
| MTBC *gyrB* gene forward primer | 0.01 μM |
| MTBC *gyrB* gene reverse primer | 0.08 μM |
| MTBC *gyrB* *N*-Cy5 probe | 0.005 μM |
| MTBC *rpoB* gene forward primer | 0.1 μM |
| MTBC *rpoB* gene reverse primer | 0.8 μM |
| MTBC *rpoB* *P1-*FAM probe | 0.1 μM |
| MTBC *rpoB* *P2-*VIC probe | 0.1uM |
| MTBC *rpoB* *P3-*ROX probe | 0.1uM |
| Mycobacterial *16S rRNA* gene reverse primer | 0.2 μM |
| Mycobacterial *16S rRNA* gene reverse primer | 1.6 μM |
| Mycobacterial *16S rRNA P-Cy5 probe* | 0.2 μM |
| Human *RPP30* gene forward primer | 0.1 μM |
| Human *RPP30* gene reverse primer | 0.8 μM |
| Human *RPP30*-CY5 probe | 0.05 μM |

**Table S2 Clinical and reference strains used in this study.**

| Bacterial species | Source | Results of MeltPlus MTB-NTM/RIF | | |
| --- | --- | --- | --- | --- |
|  |  | 16S rRNA | IS6110+gyrB | rpoB |
| *Tuberculosis* |  |  | + | + |
| *Mycobacterium tuberculosis* | Laboratory | + | + | + |
| *Mycobacterium tuberculosis* | Laboratory | + | + | + |
| *Mycobacterium tuberculosis* | Laboratory | + | + | + |
| *Mycobacterium tuberculosis* | Laboratory | + | + | + |
| *Mycobacterium tuberculosis* | Laboratory | + | + | + |
| NTM |  |  |  |  |
| *Mycobacterium avium* | Reference strain | + | - | - |
| *Mycobacterium terrae* | Reference strain | + | - | - |
| *Mycobacterium scrofulaceum* | Reference strain | + | - | - |
| *Mycobacterium abscessus* | Reference strain | + | - | - |
| *Mycobacterium shimoidei* | Reference strain | + | - | - |
| *Mycobacterium asiaticum* | Reference strain | + | - | - |
| *Mycobacterium phlei* | Reference strain | + | - | - |
| *Mycobacterium kansasii* | Reference strain | + | - | - |
| *Mycobacterium gordonae* | Reference strain | + | - | - |
| *Mycobacterium fortuitum* | Reference strain | + | - | - |
| Others respiratory pathogens |  |  |  |  |
| *Streptococcus pneumoniae* | Laboratory | - | - | - |
| *Klebsiella pneumoniae* | Laboratory | - | - | - |
| *Nocardia brasiliensis* | Reference strain | - | - | - |
| *Legionella pneumophila* | Laboratory | - | - | - |
| *Bordetella pertussis* | Laboratory | - | - | - |
| *Staphylococcus aureus* | Reference strain | - | - | - |

**Table S3. Detailed Ct value information and of the NTM-positive samples**

| Sample No. | Real-time PCR | Meltplus TB-NTM/RIF | Sample No. | Real-time PCR | Meltplus TB-NTM/RIF |
| --- | --- | --- | --- | --- | --- |
| 17 | 29.35 | NTM | 251 | 35.53 | NTM |
| 22 | 29.70 | NTM | 263 | 31.17 | NTM |
| 35 | 30.95 | NTM | 275 | 30.74 | NTM |
| 40 | 20.32 | NTM | 287 | 32.27 | NTM |
| 65 | 23.03 | NTM | 321 | 31.13 | NTM |
| 81 | 36.88 | MTBC | 338 | 31.04 | NTM |
| 105 | 31.77 | NTM | 341 | 30.46 | NTM |
| 109 | 38.11 | MTBC | 362 | 31.08 | NTM |
| 125 | 31.19 | NTM | 371 | 31.27 | NTM |
| 134 | 36.74 | NTM | 373 | 32.10 | NTM |
| 152 | 25.69 | NTM | 380 | 36.16 | NTM |
| 179 | 30.76 | NTM | 382 | 28.32 | NTM |
| 183 | 28.01 | NTM | 397 | 37.42 | MTBC |
| 206 | 31.69 | NTM | 426 | 35.43 | NTM |
| 217 | 32.61 | NTM | 441 | 31.83 | NTM |
| 235 | 35.16 | NTM | 469 | 35.53 | NTM |
| 246 | 36.25 | NTM | 475 | 26.77 | NTM |
